# Occupational Burnout Perception Scale for Nurses: A Scale Development Study Using Mixed Methods

**DOI:** 10.1155/jonm/3292864

**Published:** 2026-07-21

**Authors:** Zafer Yildiz, Sena Nur Yapar

**Affiliations:** ^1^ Faculty of Education, Sivas Cumhuriyet University, Sivas, Türkiye, cumhuriyet.edu.tr; ^2^ Institute of Health Sciences, Sivas Cumhuriyet University, Sivas, Türkiye, cumhuriyet.edu.tr

**Keywords:** burnout, mixed methods, nursing, scale

## Abstract

**Background:**

Occupational burnout experienced by nurses can negatively affect patient safety and quality of care by contributing to problems such as adverse events, malpractice and healthcare‐associated infections. Burnout can lead to negative consequences for nurses, patients, healthcare services and society.

**Objective:**

This study aims to develop a scale using mixed methods to assess nurses’ perceptions of occupational burnout.

**Methods:**

In the qualitative phase, 25 nurses were interviewed, and the scale was subsequently administered to a sample of 250 nurses for exploratory factor analysis. Four weeks later, the scale was readministered to 100 nurses selected from the same sample for confirmatory factor analysis and the assessment of test–retest reliability. Data were collected using the ‘Qualitative Data Collection Form’ and the ‘Occupational Burnout Perception Scale for Nurses’. After the qualitative data were transcribed by the researchers, themes and codes were identified. The SPSS 23.0 software package was used to analyse the quantitative data.

**Results:**

Through content analysis, five themes and 14 codes were identified. A 25‐item scale was developed based on the themes and codes identified. Through exploratory factor analysis, five items with low factor loadings and cross‐loadings were removed from the scale. The analysis resulted in a five‐factor structure consisting of 20 items.

**Conclusions:**

The scale developed through a mixed‐methods approach is recommended for use in studies evaluating nurses’ perceptions of occupational burnout.

**Implications for Nursing Leadership:**

Furthermore, the developed scale may serve as a practical tool for nurse leaders to routinely monitor burnout levels, identify nurses at risk at an early stage and plan management strategies that promote staff well‐being.

## 1. Introduction

Burnout is one of the key issues that affect both individuals and institutions in professional life. Burnout syndrome is fundamentally a chronic response to workplace stress that diminishes both individual and professional satisfaction. It affects the individual across all domains, including physical, psychological and emotional dimensions [1]. The prevalence of burnout syndrome, which was defined by Herbert Freudenberger as a state of stress experienced by individuals working in close contact with others, has increased in line with rising workplace demands [2].

Maslach and Jackson (1981) proposed one of the most systematic approaches to the conceptualisation of burnout. They examined burnout across three dimensions: emotional exhaustion, depersonalisation and reduced personal accomplishment. Emotional exhaustion is defined as a feeling of being overextended and depleted due to work; depersonalisation is defined as the development of negative and excessively detached attitudes towards those receiving services; and reduced personal accomplishment is defined as a sense of inadequacy and lack of achievement in one’s work [3]. The scale developed for this study was based on Maslach’s three‐dimensional model. However, in the context of nursing, burnout is not limited to psychological dimensions. Organisational and professional contextual factors are integral to this process [4]. Accordingly, the model was adapted to take into account contextual factors specific to the nursing work environment.

The World Health Organisation (WHO) defines burnout as a syndrome resulting from chronic workplace stress that has not been successfully managed. The WHO has stated that burnout is characterised by dimensions such as energy depletion and fatigue, increased mental distance from one’s job, negative or cynical attitudes towards it and a sense of ineffectiveness and lack of accomplishment [5]. Burnout is particularly common among healthcare professionals who are in close and frequent contact with others [6]. Burnout is a gradual process characterised by physical, social and psychological exhaustion [7].

Work‐related stress, without effective coping mechanisms, leads to burnout when it exceeds the individual’s capacity for adaptation [8]. Another definition of burnout is the depletion of mental and physical energy. Burnout manifests in individuals as emotional exhaustion, desensitisation and a reduced sense of personal achievement [9]. It is known that nurses are the group among healthcare workers who experience the highest levels of burnout [6].

In recent years, burnout syndrome has become increasingly prevalent among nurses [10]. Getie et al. (2025), in a study examining the global prevalence of nurse burnout, reported that 33% of nurses experienced emotional exhaustion, 25% experienced depersonalisation and 33% experienced reduced personal accomplishment [4]. Nurses experiencing burnout may exhibit emotional exhaustion, lack of empathy, decreased self‐esteem and reduced self‐confidence [10]. Nurses working in a busy environment begin to experience a loss of motivation and satisfaction due to personal and organisational factors. This situation can become even more severe, resulting in hopelessness, emotional detachment and disillusionment [7].

Burnout among nurses is a serious occupational problem that affects patients and society. High levels of burnout negatively affect nurses individually and impose additional costs on healthcare systems [6]. Burnout among nurses also poses significant threats to patient safety and quality of care. Burnout is an independent predictor of adverse events, medical errors, healthcare‐associated infections and malpractice [11].

Burnout is influenced by many factors, including workload, values, professionalism, gender, genetics and the value placed on patient care [9, 12]. Factors such as the increasing complexity of healthcare services, growing competition and rising expectations also make it difficult for nurses to adapt to their working environment [6]. In addition to these factors, the increased electronic documentation, difficulties in scheduling work hours and administrative issues also contribute to burnout [12].

The increasing prevalence of burnout in nursing poses a threat to healthcare services and patient safety. This threat stems from nurses losing interest in their work. Nurses experiencing burnout show impairments in clinical decision‐making and reaction times, as well as weakened critical thinking skills. In addition, nurses may experience absenteeism and presenteeism [13].

Considering the individual and social consequences that burnout among nurses can cause, early identification and intervention are of great importance [14]. Burnout is a subjective process grounded in how individuals perceive and experience chronic work‐related stress. In this context, assessing perceptions of burnout is considered an effective tool for early awareness and intervention [3, 5]. A review of the literature reveals that the scales used to assess burnout in nursing are generic scales covering many occupational groups [15, 16]. An examination of burnout scales developed for nurses indicates that they are generally tailored to nurses working within specific clinical domains [17, 18]. Accordingly, it is thought that the ‘Occupational Burnout Perception Scale for Nurses’, developed using mixed methods in this study, will have positive effects on the early identification of burnout that may develop in nurses and on the planning of necessary interventions. It is anticipated that the study will contribute to the literature, given that the developed scale is specific to the nursing profession, is applicable to all nurses working in shift‐work systems, and that such studies remain limited in the existing literature [17, 19].

## 2. Method

### 2.1. Type of Study

The study aimed to develop a scale using mixed methods to evaluate nurses’ perceptions of occupational burnout. An exploratory sequential mixed‐methods design was used in the study. The first phase of the study consisted of qualitative analysis, while the second phase consisted of quantitative analysis.

### 2.2. Study Population and Sample

The study group for the qualitative and quantitative data collection phases consisted of nurses working at Sivas Numune Hospital. Interviews with nurses were conducted outside the hospital. Convenience sampling and maximum variation sampling were used in the qualitative part of the study. Data collection continued until data saturation was achieved and no new themes emerged [20]. Accordingly, 25 nurses were included in the qualitative data collection phase of the study. In validity and reliability studies, a sample size of ten times the number of items is considered sufficient for determining the sample size [21, 22]. In this context, 250 nurses were included in the study because the scale contained 25 items during the quantitative data collection process. Four weeks after the initial administration, the scale was readministered to 100 nurses selected from the same sample, and confirmatory factor analysis (CFA) and test–retest reliability were assessed. The qualitative data collection process was conducted between 15 June and 30 June 2024, while the quantitative data collection process was conducted between 15 July and 15 October 2024.

### 2.3. Ethical Considerations

To conduct the study, permission was obtained from the Cumhuriyet University Non‐Interventional Clinical Research Ethics Committee with approval number 2024–05/17. Participants were informed that their data would be kept confidential and used only for the purposes of this study. The principles of the Declaration of Helsinki were followed in the study. Written informed consent was obtained from all participants.

### 2.4. Inclusion and Exclusion Criteria

Nurses who had been working as nurses for at least four years, who worked shift patterns (mixed day and night shifts) and who agreed to participate after being informed about the study were included in the study. Nurses holding managerial positions were excluded.

### 2.5. Data Collection Tools

1. Qualitative data collection form: In the qualitative data collection phase of the study, six open‐ended questions prepared by the researchers were used to explore nurses’ perceptions of occupational burnout (e.g., Evaluate the difficulties you experience in your professional life; What are the effects of these difficulties on you? What is burnout and what are its effects on individuals?). The questions were designed to allow nurses to express their perceptions of burnout [23, 24].
2. Occupational burnout perception scale for nurses: The scale developed by the researchers based on qualitative data consists of 25 items.

### 2.6. Study Process

#### 2.6.1. Qualitative Phase

- In the initial phase, structured interviews incorporating open‐ended questions were conducted with 25 nurses who had been working in shift‐work systems for at least 4 years across different hospital units. During the interviews, a total of six questions were posed regarding the challenges experienced in professional practice, the impacts of these challenges, burnout and attitudes towards the profession. Prior to data collection, informed consent was obtained from the nurses, and the responses were audio‐recorded.
- The audio recordings were subsequently transcribed by the researchers. Frequently used words and expressions identified in the interviews were categorised into groups, and themes and codes were derived accordingly.

#### 2.6.2. Quantitative Phase

- In the study, the scale items were developed based on themes and codes derived from qualitative data reflecting the experiences and perspectives of nurses actively working in the field. The aim was to construct a measurement instrument that reflects the real‐life experiences of the target population. Therefore, the item pool was generated directly from qualitative data obtained from nurses. Expert review was not conducted during this stage [25, 26].
- The generated items were initially administered to a sample of 250 participants, and the resulting data were subjected to exploratory factor analysis.
- Four weeks after the initial administration, the scale was readministered to a sample of 100 participants with similar professional characteristics to assess test–retest reliability. The data obtained were subsequently subjected to CFA.

### 2.7. Data Analysis

The notes taken during the qualitative interviews were transcribed by the researcher, and themes and codes were identified accordingly. Content analysis was used to evaluate the qualitative data obtained during the data collection process. Factor analysis was performed using SPSS 23.0 and SPSS AMOS 23.0 software to analyse the quantitative data. The suitability of exploratory factor analysis was assessed using Bartlett’s test of sphericity and the Kaiser–Meyer–Olkin (KMO) measure. The Varimax rotation method was used in the exploratory factor analysis. Regression weights and coefficients were calculated using CFA established within a structural equation modelling framework. Test–retest reliability was assessed using Pearson correlation coefficients between the scores obtained from the first and second administrations.

## 3. Findings From the Qualitative Data Analysis

Table 1 presents the professional characteristics of the nurses who participated in the qualitative phase of the study. Of the participants, 76% were female, 44% were between 31 and 39 years of age, 36% worked in internal medicine units and 36% had 9–13 years of professional experience.

**TABLE 1:** Professional characteristics of the qualitative data participants.

Professional characteristics	Groups	*n*: 25	%
Gender	Female	19	76
	Male	6	24

Age groups	≤ 30	9	36
	31–39	11	44
	≥ 40	5	20

Clinical units	Intensive care units	6	24
	Internal medicine units	9	36
	Emergency units	5	20
	Surgical units and operating rooms	5	20

Years of experience	4–8 years	8	32
	9–13 years	9	36
	14 years and above	8	32

Table 2 presents the themes and codes obtained from the qualitative data analysis. Qualitative analysis revealed five themes: ‘Lack of motivation’, ‘Hopelessness’, ‘Worthlessness’, ‘Exhaustion’ and ‘Desensitisation’.

**TABLE 2:** Themes and codes.

Themes	Codes	Frequency
Lack of motivation	Loss of motivation	47
	Unhappiness	30

Hopelessness	Irritability	32
	Fatigue	27
	Stress	13

Worthlessness	Feeling insignificant	90
	Injustice	26
	Professional solidarity	19
	Workplace mobbing	15

Exhaustion	Negative effects	62
	Role ambiguity	38
	Sleep disturbance	7

Desensitisation	Lack of empathy	24
	Feelings of inadequacy	13

### 3.1. Lack of Motivation

The codes for the theme of lack of motivation were identified as ‘Loss of Motivation’ and ‘Unhappiness’. Some examples of statements related to these codes are presented below:

 Nurse 1: ‘Once the excitement we feel about our profession wears off, we continue to do our job out of obligation’.
 Nurse 2: ‘My motivation is zero when I’m working. Due to my low motivation, I am unable to provide the necessary care to individuals’.
 Nurse 12: ‘Burnout, in my opinion, is feeling unhappy at work. There is no other meaning to it’.

### 3.2. Hopelessness

The codes for the theme of hopelessness were identified as ‘Irritability’, ‘Fatigue’ and “Stress’. Some examples of statements related to these codes are presented below:

 Nurse 4: ‘Exhausted individuals inevitably become tense and restless in the workplace. In particular, tense nurses may get into arguments with patients and their relatives’.
 Nurse 8: ‘When I get home, I feel extremely tired. I don’t even want to eat; I just want to put my feet up and relax’.
 Nurse 4: ‘I come to work feeling stressed. I even started feeling stressed the day before my 24‐h shift. Even when I leave work and go home, I still feel like I’m at the hospital’.

### 3.3. Worthlessness

The codes for the theme of worthlessness were identified as ‘Feeling Insignificant’, ‘Injustice’, ‘Professional Solidarity’ and ‘Workplace Mobbing’. Some examples of statements related to these codes are presented below:

 Nurse 4: ‘I do not believe that nurses are valued in any way’.
 Nurse 2: ‘The management is not giving credit where credit is due. This situation is having a negative impact on me’.
 Nurse 2: ‘The problems I experience with my colleagues in the workplace are really exhausting me. Individuals who have reached a certain level of seniority look for my weaknesses and try to make me feel inadequate’.
 Nurse 12: ‘My position was changed without explanation. This made me question my work. I started to wonder whether I was performing poorly’.

### 3.4. Exhaustion

The codes for the theme of exhaustion were identified as ‘Adverse Effects’, ‘Role Ambiguity’ and ‘Sleep Disturbance’. Some examples of statements related to these codes are presented below:

 Nurse 6: ‘The difficulties I experience in my job are negatively affecting my physical health’.
 Nurse 11: ‘We generally spend more time at the hospital than in our social lives. We cannot devote enough time to our spouses and children. Sometimes we cannot even find time for ourselves’.
 Nurse 5: ‘All the work we do outside of our own duties is actually one of the main causes of the difficulties we face’.
 Nurse 10: ‘We don’t have a sleep routine. For me, the biggest problem is a disrupted sleep routine’.

### 3.5. Desensitisation

The codes for the theme of desensitisation were identified as ‘Lack of Empathy’ and ‘Feeling Inadequate’. Some examples of statements related to these codes are presented below:

 Nurse 11: ‘Death seems normal to us now. This is a very bad thing. Even when your own family members are ill, you do not become overly concerned unless they are hospitalised or admitted to intensive care’.
 Nurse 12: ‘We tend not to use our empathy skills. You get used to it; you have to get used to it somehow. Rather than seeing elderly people suffer, you end up saying that it’s better they have passed away’.
 Nurse 14: ‘When I feel like I can’t cope, I feel exhausted. Even checking the patient’s temperature feels like a burden at that moment’.

## 4. Findings From the Quantitative Data Analysis

### 4.1. Exploratory Factor Analysis Findings

Table 3 presents the professional characteristics of the participants included in the exploratory factor analysis. Of the participants, 72% were female, 38% were aged 30 years or younger, 31.4% worked in internal medicine units and 36.8% had 4–8 years of professional experience.

**TABLE 3:** Professional characteristics of participants included in the exploratory factor analysis.

Professional characteristics	Groups	*n*: 250	%
Gender	Female	180	72
	Male	70	28

Age groups	≤ 30	95	38
	31–39	80	32
	≥ 40	75	30

Clinical units	Intensive care units	76	30.4
	Internal medicine units	78	31.2
	Emergency units	50	20
	Surgical units and operating rooms	46	18.4

Years of experience	4–8 years	92	36.8
	9–13 years	81	32.4
	14 years and above	77	30.8

Table 4 shows the results of Bartlett’s test of sphericity and the KMO measure conducted to assess the suitability of the data for exploratory factor analysis. The KMO coefficient of 0.859 indicates that the sample size is adequate. Bartlett’s test of sphericity showed that the data were suitable for factor analysis (*p* < 0.001). These findings indicate that the correlation matrix was factorable and that the data were appropriate for exploratory factor analysis.

**TABLE 4:** Suitability of the data for exploratory factor analysis.

KMO measure of sampling adequacy	0.859	
Bartlett’s test of sphericity	Chi‐square value	2237
	*p* value: 0.001	0.000

Abbreviation: KMO, Kaiser–Meyer–Olkin.

The Varimax rotation method was used in the exploratory factor analysis. Based on the analysis, items with factor loadings below 0.5 were removed from the scale. After removing items with low factor loadings, 20 items remained on the scale. A five‐factor structure was obtained in the exploratory factor analysis of the 20‐item scale. Table 5 presents the total explained variance of the scale. The variance explained by the first factor is 15.896, followed by 14.523 for the second factor, 12.067 for the third factor, 11.681 for the fourth factor and 9.790 for the fifth factor. The total variance explained is 63.957%.

**TABLE 5:** Total explained variance of the scale.

Scale subdimension	Items	Factor loading value	Variance	Cumulative variance (%)
Factor 1—Lack of motivation	Q9	0.743	15.896	15.896
	Q6	0.709		
	Q8	0.698		
	Q14	0.667		
	Q7	0.663		

Factor 2—Hopelessness	Q12	0.801	14.523	30.419
	Q13	0.737		
	Q15	0.728		
	Q24	0.699		

Factor 3—Worthlessness	Q20	0.817	12.067	42.486
	Q21	0.773		
	Q22	0.627		
	Q25	0.538		

Factor 4—Exhaustion	Q2	0.799	11.681	54.167
	Q4	0.734		
	Q3	0.631		
	Q1	0.542		

Factor 5—Desensitisation	Q11	0.866	9.790	63.957
	Q10	0.660		
	Q16	0.626		

*Note:* Q: question.

### 4.2. Confirmatory Factor Analysis Findings

Table 6 presents the professional characteristics of the participants included in the CFA. Of the participants, 68% were female, 51% were aged 30 years or younger, 30% worked in intensive care units and 37% had 4–8 years of professional experience.

**TABLE 6:** Professional characteristics of participants included in the confirmatory factor analysis.

Professional characteristics	Groups	*n*: 100	%
Gender	Female	68	68
	Male	32	32

Age groups	≤ 30	51	51
	31–39	30	30
	≥ 40	19	19

Clinical units	Intensive care units	30	30
	Internal medicine units	31	31
	Emergency units	20	20
	Surgical units and operating rooms	19	19

Years of experience	4–8 years	37	37
	9–13 years	32	32
	14 years and above	31	31

Table 7 presents the regression weights obtained from the CFA conducted using structural equation modelling. Regression weights indicate the strength of the relationship between observed variables and latent variables, that is, that is, factor loadings. Since *p* < 0.05 for all regression weights in the table, the factor loadings are significant. The significance of the factor loadings indicates that the items are adequately loaded onto their respective factors [27].

**TABLE 7:** Regression weights related to the model.

Direction of relationship	Estimate	Standard error	Critical ratio	*p*
Q9 ⟵ F1	1.000			
Q6 ⟵ F1	0.622	0.068	9.285	0.001
Q8 ⟵ F1	0.854	0.069	12.462	0.001
Q14 ⟵ F1	0.936	0.072	12.955	0.001
Q7 ⟵ F1	0.681	0.067	10.152	0.001
Q12 ⟵ F2	1.000			
Q13 ⟵ F2	1.209	0.076	15.856	0.001
Q15 ⟵ F2	0.684	0.068	10.004	0.001
Q24 ⟵ F2	0.751	0.074	10.193	0.001
Q20 ⟵ F3	1.000			
Q21 ⟵ F3	1.634	0.152	10.733	0.001
Q22 ⟵ F3	0.506	0.071	7.122	0.001
Q25 ⟵ F3	0.998	0.124	8.061	0.001
Q2 ⟵ F4	1.000			
Q4 ⟵ F4	0.673	0.090	7.515	0.001
Q3 ⟵ F4	0.613	0.072	8.491	0.001
Q1 ⟵ F4	0.936	0.104	8.971	0.001
Q11 ⟵ F5	1.000			
Q10 ⟵ F5	1.070	0.142	7.510	0.001
Q16 ⟵ F5	1.149	0.157	7.323	0.001

*Note:* Q: question, F: factor.

The obtained factor loadings indicate that the items are strongly associated with the latent variables they represent. In particular, the high factor loadings of certain items (Q8, Q14 and Q13) indicate that these items are the strongest indicators of their respective factors. Conversely, the relatively lower factor loadings of items such as Q22 and Q3 suggest that their association with the relevant factors is more limited than that of the other items; however, these values remain within acceptable limits. Additionally, the high critical ratio values for all items support the stability of the parameter estimates and the adequacy of the model. These findings indicate that the measurement model is well aligned with the data and that the factor structure has been confirmed. Accordingly, it can be concluded that the items included in the model adequately represent the theoretically defined factors and that the construct validity of the scale is supported.

Table 8 presents the average variance extracted (AVE) values for the scale. An examination of the AVE values of the subdimensions indicates that some factors have AVE values below 0.50. However, given that these values are above 0.40 and that the factor loadings and internal consistency coefficients are at acceptable levels, the findings provide partial support for convergent validity and should be interpreted with caution [28].

**TABLE 8:** Average variance extracted (AVE) values of the scale.

Factor	Number of items	AVE
Factor 1 – Lack of Motivation	5	0.533
Factor 2 – Hopelessness	4	0.603
Factor 3 – Worthlessness	4	0.444
Factor 4 – Exhaustion	4	0.425
Factor 5 – Desensitisation	3	0.427

**FIGURE 1:**
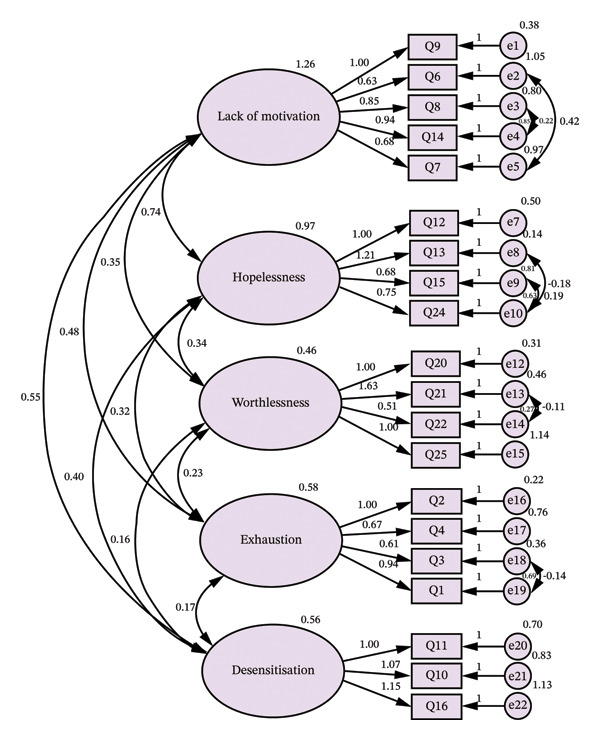
Confirmatory factor analysis model. CMIN/df: 2.077; TLI: 0.903; IFI: 0.923; CFI: 0.922; RMSEA: 0.066; GFI: 0.890; AGFI: 0.850. Abbreviations: Q: question; e: error.

Figure 1 presents the CFA model of the Occupational Burnout Perception Scale for Nurses. The model consists of five factors: Lack of Motivation, Hopelessness, Worthlessness, Exhaustion and Desensitisation. The standardised factor loadings of the items on their respective factors were found to be at acceptable levels. Furthermore, the model fit indices indicated that the values of CMIN/df = 2.077, TLI = 0.903, IFI = 0.923, CFI = 0.922, RMSEA = 0.066, GFI = 0.890 and AGFI = 0.850 were within acceptable fit ranges [29, 30]. These findings support the five‐factor structure identified through exploratory factor analysis and demonstrate that the model shows an acceptable fit to the data.

The fit indices show that the model demonstrates an acceptable fit to the data. Thus, the CFA findings provide evidence for the construct validity of the five‐factor structure revealed by exploratory factor analysis. The construct validity of the scale has been supported by CFA. Together with the reliability analyses, these findings suggest that the developed scale may be a useful instrument for measuring the level of occupational burnout among nurses.

### 4.3. Explanation of the Modification Requirements

Table 9 shows the modification indices between error terms associated with the items and questions in the CFA model. Pairs with high modification indices may reflect similarities in item content. Allowing covariance between the error terms of two items in the model contributes to a better‐fitting model.

**TABLE 9:** Parameter pairs with high modification indices.

Error items	Questions	Modification indices
e2 ⟶ e5	Q6 ⟶ Q7	42.488
e3 ⟶ e4	Q8 ⟶ Q14	14.354
e7 ⟶ e9	Q13 ⟶ Q24	15.257
e8 ⟶ e9	Q15 ⟶ Q24	25.417
e11 ⟶ e12	Q21 ⟶ Q22	4.418
e16 ⟶ e17	Q3 ⟶ Q1	7.598

*Note:* Q: questions, e: error.

Questions 6 and 7 reflect negative experiences, such as a lack of motivation and communication problems in the work environment. Questions 8 and 14 reflect questioning the purpose of the profession and the desire to leave the profession. Questions 15 and 24 reflect fatigue and energy loss, and Questions 21 and 22 reflect mobbing and perceived injustices in the work environment. The listed items are thematically related and may be perceived similarly by participants.

Questions 13 and 24 reflect stressful work and energy loss, while Questions 3 and 1 reflect negative effects on social life and performing duties outside participants’ job responsibilities. Although the items appear to have different content, the high modification indices suggest that participants may perceive them as having commonalities.

Table 10 presents the Cronbach’s alpha values for the scale factors and the overall scale. An examination of the table indicates that the overall internal consistency coefficient of the scale is quite high, suggesting that the scale demonstrates very good reliability. Regarding the subdimensions, the Lack of Motivation and Hopelessness factors exhibit high levels of internal consistency, whereas Worthlessness, Exhaustion and Desensitisation demonstrate acceptable levels of internal consistency.

**TABLE 10:** Cronbach’s alpha coefficients for the scale factors and total score.

Factors	Number of items	Cronbach’s alpha
Factor 1—lack of motivation	5	0.847
Factor 2—hopelessness	4	0.846
Factor 3—worthlessness	4	0.725
Factor 4—exhaustion	4	0.710
Factor 5—desensitisation	3	0.712
Total	**20**	**0.909**

*Note:* The bold values indicate the total number of items in the scale and the overall Cronbach’s alpha coefficient.

Table 11 presents the correlations between the corresponding factor scores obtained from the exploratory and confirmatory factor analyses. The relationship between the corresponding factor scores obtained from the exploratory and confirmatory factor analyses was examined. Each factor was correlated with its corresponding factor, and the Pearson correlation coefficient was calculated. In all pairwise comparisons, the *p* values were found to be less than 0.01, indicating that the relationships were statistically significant. Based on these findings, the scale demonstrates good test–retest reliability.

**TABLE 11:** Correlation between exploratory and confirmatory factor analyses.

Factors	Pearson correlation	*p*
Factor 1—lack of motivation	0.896^∗∗^	0.001
Factor 2—hopelessness	0.875^∗∗^	0.001
Factor 3—worthlessness	0.833^∗∗^	0.001
Factor 4—exhaustion	0.908^∗∗^	0.001
Factor 5—desensitisation	0.915^∗∗^	0.001

^∗∗^ *p* < 0.01.

Table 12 presents the Guttman split‐half reliability coefficients of the scale. According to the reliability analysis based on the split‐half method, the Cronbach’s alpha coefficient for the first half was 0.883, indicating a high level of internal consistency. The Cronbach’s alpha value for the second half was 0.736, reflecting an acceptable level of internal consistency [31]. These findings suggest that the scale is generally reliable; however, some differences in internal consistency were observed between the two halves.

**TABLE 12:** Guttman split‐half reliability values of the scale.

Parts	Items	Cronbach’s alpha
Part 1	Q9, Q6, Q8, Q14, Q7, Q12, Q13, Q15, Q24, Q20	0.883
Part 2	Q21, Q22, Q25, Q3, Q4, Q1, Q2, Q11, Q10, Q16	0.736

*Note:* Q: question.

## 5. Integration of Qualitative and Quantitative Data

Themes and codes were identified through qualitative data analysis. Items for the scale were created based on the codes obtained from the qualitative data analysis. The main objective was to ensure that the responses given by participants reflected their emotional and cognitive experiences and could be meaningfully represented in quantitative form. In this regard, the following connections are noteworthy based on the codes obtained.

The theme of Lack of Motivation includes the codes ‘loss of motivation’ and ‘unhappiness’. Nurses who identified with the theme of lack of motivation spoke about questioning their profession, considering leaving it, their level of motivation at work, their feelings while working and the communication problems they experienced. In this regard, items such as ‘I feel unhappy while working’, ‘I do not want to communicate with anyone in the work environment’, ‘I question my reasons for remaining in this profession’, ‘I do not want to continue in this job’ and ‘I feel the work environment reduces my motivation’ were added to the scale.

Under the theme of Hopelessness, the codes ‘irritability’, ‘fatigue’ and ‘stress’ were identified. Nurses associated with this theme described negative emotions such as tension and stress experienced during work, as well as physical and psychological effects such as decreased energy and fatigue. Accordingly, the items ‘I feel stressed while working’, ‘I feel tense while working’, ‘I consistently feel tired’ and ‘I feel depleted of energy’ were added to the scale.

Under the theme of Worthlessness, the codes ‘feeling insignificant’, ‘injustice’, ‘professional solidarity’ and ‘workplace mobbing’ were identified. Nurses associated with this theme discussed workplace injustice, mobbing and the lack of professional solidarity and recognition. In line with this, the following items were added to the scale: ‘I am worn down by injustices in the workplace’, ‘I am worn down by mobbing in the workplace’, ‘I think we are not valued as we deserve in our profession’ and ‘I am worn down by not working in solidarity with my colleagues’.

The theme of Exhaustion includes the codes ‘adverse effects’, ‘role ambiguity’ and ‘sleep disturbance’ were identified. Nurses associated with this theme described the negative impacts of the profession on their physical health and social life, as well as problems arising from role ambiguity. In this regard, the following items were added to the scale: ‘I think the nursing profession has a negative impact on my physical health’, ‘My work schedule disrupts my sleep pattern’, ‘Performing duties outside my job responsibilities wears me out’ and ‘I think the nursing profession has a negative impact on my social life’.

Under the theme of Desensitisation, the codes ‘Lack of Empathy’ and ‘Feelings of Inadequacy’ were identified. Nurses associated with this theme described reduced empathy towards patients, feelings of inadequacy in their professional roles and emotional detachment from patient care. Accordingly, the items ‘I feel that I am not feel committed to my work’, ‘I feel inadequate while working’ and ‘I feel I have become desensitised towards patients’ were added to the scale.

### 5.1. Scoring of the Scale

The scale developed in this study to assess nurses’ perceptions of occupational burnout consists of 20 items in its final version. Each item is rated on a 5‐point Likert scale. The scale, which demonstrated acceptable validity and reliability values, consists of five factors. In the final version of the scale, the Lack of Motivation factor consists of items 5, 6, 7, 8 and 13; the Hopelessness factor consists of items 11, 12, 14 and 19; the Worthlessness factor consists of items 16, 17, 18 and 20; the Exhaustion factor consists of items 1, 2, 3 and 4; and the Desensitisation factor consists of items 9, 10 and 15. An increase in the score obtained from the scale indicates an increase in the individual’s level of burnout. When analysing the scale, researchers can analyse both mean and total scores.

## 6. Discussion

In this study, a scale was developed to measure the perceived level of occupational burnout among nurses using a mixed‐methods approach, based on the views and experiences of the target population. Accordingly, a five‐factor scale consisting of 20 items was developed. The scale includes items addressing the effects of occupational burnout on individuals, as well as items evaluating nurses’ perceptions of occupational burnout.

The burnout model developed by Maslach and Jackson (1981), along with the Maslach Burnout Inventory, is widely used in the assessment of burnout. The scale was developed for application to a broad range of human service professionals, including nurses, and thus functions as a general measurement instrument. However, its inability to adequately reflect the profession‐specific context of burnout constitutes one of its key limitations [32].

The scale developed by Teymori et al. (2024) is specific to operating room nurses and is limited to the surgical setting, focussing on the causes of burnout. In contrast, the scale developed in the present study has a broader scope and can be applied to all nurses working in shift‐work systems. Furthermore, by focussing on the causes of burnout, the scale developed by Teymori et al. assesses individuals’ perceptions of factors leading to burnout rather than directly measuring the level of burnout itself [19]. Accordingly, the scale developed in this study addresses a different dimension by focussing on the outcomes of burnout and its effects on the individual.

In the study conducted by Choudhary et al. (2022) to assess burnout syndrome among intensive care nurses, a multidimensional scale was developed. An examination of the items indicates that they include statements reflecting the impact of burnout on individuals. However, the study was conducted solely with intensive care nurses and therefore included a more limited sample. Although the dimensions in the scale’s factor structure were not clearly and conceptually defined, an examination of the item content suggests that the scale includes themes consistent with the burnout model proposed by Maslach [17]. In this respect, although both scales are based on the burnout model proposed by Maslach, the present study extends the model within the nursing context and addresses burnout through more comprehensive dimensions. Additionally, by selecting a sample encompassing a more diverse group of nurses, the study aimed to enhance the generalisability of the findings.

In the study conducted in Taiwan by Chen et al. (2026), a scale was developed to assess burnout among nurses and its psychometric properties were examined using secondary data. A multidimensional structure was adopted, resulting in a scale comprising nine factors. An examination of the scale items indicates that some items are similar to those developed in the present study. However, while Chen et al. conceptualised burnout in broader dimensions [33], the present study differs by focussing on individuals’ perceptions of occupational burnout and its effects on the individual.

İsmail et al. (2025) developed a burnout assessment instrument for healthcare professionals and examined its psychometric properties. The study yielded a two‐dimensional structure comprising cognitive and emotional burnout. However, the scale provides a general framework applicable to all healthcare professionals and reflects the profession‐specific contextual characteristics of nursing only to a limited extent [34]. The scale developed in this study was constructed based on qualitative data derived directly from the experiences of nurses actively working in the field and addresses both the effects of occupational burnout on individuals and nurses’ perceptions of occupational burnout in a more comprehensive manner. Furthermore, its structure, comprising a greater number of factors, allows for a more detailed evaluation of the multidimensional nature of burnout within the nursing context.

The early identification of occupational burnout among nurses is of critical importance for maintaining individual well‐being and ensuring the continuity of care quality. In this regard, the scale developed is expected to make a practical contribution to nursing management. The scale may enable managers to regularly assess burnout levels, identify nurses at risk and support the planning of appropriate interventions. It may serve as a guide for developing strategies to address factors that can contribute to burnout, such as workload regulation, shift scheduling, psychosocial support and team communication. From a health policy perspective, it may also facilitate addressing burnout at the institutional level, thereby providing a basis for planning and implementing initiatives aimed at promoting and protecting employee health. In this context, the scale can be regarded not only as an individual assessment tool but also as an instrument that can contribute to organisational development and quality improvement processes [3, 12, 14, 24].

A review of the literature indicates that scales developed to measure burnout tend to focus on specific professional groups or clinical settings, while others address burnout within a more general framework. It is noteworthy that a substantial proportion of existing scales focus on the causes or standard dimensions of burnout and remain limited in capturing the effects experienced by nurses and their perceptions of occupational burnout [17, 19, 32–34]. The scale developed within the scope of this study was constructed based on qualitative data derived from the direct experiences of nurses and focuses on the effects of occupational burnout on the individual and nurses’ perceptions of occupational burnout rather than its causes. Its applicability to a broader sample of nurses working in different clinical units and shift‐work systems represents an important strength that enhances the generalisability of the scale. It is anticipated that this scale will contribute to the literature, future research and practice by enabling a more comprehensive evaluation of nurses’ perceptions of occupational burnout.

## 7. Conclusion

A limited number of occupational burnout perception scales in nursing developed by reviewing the literature were identified in the literature, and the available scales were found to assess burnout among nurses working in specific units. The scale developed in this study can be applied to nurses across all clinical settings. During the research, nurses working in a wide range of clinical units were included in the study to ensure diversity in the sample. The existing scales include items related to the causes of burnout and the personal and occupational effects of burnout [17, 19]. The scale developed in this study includes both cause‐ and effect‐related items assessing nurses’ perceptions of occupational burnout. In this regard, it is expected that the scale will contribute to the literature.

Using a mixed‐methods approach, this study aimed to develop a scale for assessing nurses’ perceptions of occupational burnout. While the item pool is often created based on expert opinions during the quantitative scale development process, the item pool in this study was developed from the experiences and views of nurses working in the field during the qualitative phase. Therefore, the scale items reflect the current experiences, feelings and perceptions of nurses more accurately within the continuously changing clinical environment. It is recommended that the developed scale be used in studies aimed at evaluating nurses’ perceptions of occupational burnout.

## 8. Implications for Nursing Management

From a nursing leadership perspective, this scale can serve not only as a tool for identifying individual levels of occupational burnout among nurses but also as an assessment instrument that supports evidence‐based managerial decision‐making. Through its regular administration, nurse leaders can identify nurses at high risk of burnout at an early stage and plan individualised support interventions. Furthermore, based on the results obtained from the scale’s subdimensions, nurse leaders can make informed decisions regarding workload distribution, shift scheduling, task allocation and workforce management, while also identifying unit‐specific burnout profiles to develop targeted interventions. In addition, the findings obtained from the scale may help identify organisational issues, such as perceptions of injustice, insufficient teamwork and workplace mobbing, at an early stage, thereby contributing to improvements in both staff well‐being and the quality of patient care. Accordingly, the developed scale may serve not only as a research instrument but also as a practical management tool that supports nursing leaders in making strategic decisions aimed at promoting staff well‐being, strengthening workforce retention and ensuring the sustainability of high‐quality patient care.

## 9. Limitations of the Study

The conduct of the study in a single centre and the inclusion of a sample drawn from the same institution—although participants were recruited from different units—limit the generalisability of the findings. Future research should consider multicentre studies with larger and more heterogeneous samples drawn from different institutions and working under diverse conditions. In addition, during the scale development process, a participant‐based approach grounded in direct experiences was adopted for item generation, and expert input was not incorporated; this may constitute a limitation in terms of content validity. During the scale development process, the item pool was generated directly from qualitative data derived from nurses’ experiences; however, expert review of the item pool was not conducted. Although this approach may enhance the contextual relevance of the items, formal assessment of content validity was not performed and should be considered a limitation of the study.

## Author Contributions

Both authors contributed to the conceptualisation and design of the study. Zafer Yildiz was responsible for the methodological planning of the study, supervision of the research process and overall supervision of the project. Sena Nur Yapar contributed to the review of nursing‐specific literature and the development of the profession‐specific content. Both authors contributed to the literature review, data collection, interpretation of the findings and preparation of the manuscript.

## Funding

No funding was received for this manuscript.

## Disclosure

All authors read and approved the final version of the manuscript.

## Conflicts of Interest

The authors declare no conflicts of interest.

## Data Availability

Research data are not shared. The datasets generated and/or analysed during the current study are available from the corresponding author on reasonable request.
